# Sport Injuries Sustained by Athletes with Disability: A Systematic Review

**DOI:** 10.1007/s40279-016-0478-0

**Published:** 2016-02-04

**Authors:** Richard Weiler, Willem Van Mechelen, Colin Fuller, Evert Verhagen

**Affiliations:** 1 Amsterdam Collaboration on Health and Safety in Sports, Department of Public and Occupational Health, EMGO Institute for Health and Care Research, VU University Medical Center, Amsterdam, The Netherlands; 2 The FA Centre for Disability Football Research, St Georges Park, Burton-Upon-Trent, Staffordshire, UK; 3 University College London Hospitals NHS Foundation Trust, London, UK; 4 Fortius Clinic, London, UK; 5 Faculty of Health and Behavioural Sciences, School of Human Movement and Nutrition Sciences, University of Queensland, Brisbane, QLD Australia; 6 School of Public Health, Physiotherapy and Population Sciences, University College Dublin, Dublin, Ireland; 7 UCT/MRC Research Unit for Exercise Science and Sports Medicine (ESSM), Department of Human Biology, Faculty of Health Sciences, University of Cape Town, Cape Town, South Africa; 8 Colin Fuller Consultancy Ltd, Sutton Bonington, UK; 9 Australian Centre for Research into Injury in Sport and its Prevention, Federation University Australia, Ballarat, VIC Australia

## Abstract

**Background:**

Fifteen percent of the world’s population live with disability, and many of these individuals choose to play sport. There are barriers to sport participation for athletes with disability and sports injury can greatly impact on daily life, which makes sports injury prevention additionally important.

**Objective:**

The purpose of this review is to systematically review the definitions, methodologies and injury rates in disability sport, which should assist future identification of risk factors and development of injury prevention strategies. A secondary aim is to highlight the most pressing issues for improvement of the quality of injury epidemiology research for disability sport.

**Methods:**

A search of NICE, AMED, British Nursing Index, CINAHL, EMBASE and Medline was conducted to identify all publications up to 16 June 2015. Of 489 potentially relevant articles and reference searching, a total of 15 studies were included. Wide study sample heterogeneity prevented data pooling and meta-analysis.

**Results:**

Results demonstrated an evolving field of epidemiology, but with wide differences in sports injury definition and with studies focused on short competitions. Background data were generally sparse; there was minimal exposure analysis, and no analysis of injury severity, all of which made comparison of injury risk and injury severity difficult.

**Conclusion:**

There is an urgent need for consensus on sports injury definition and methodology in disability sports. The quality of studies is variable, with inconsistent sports injury definitions, methodologies and injury rates, which prevents comparison, conclusions and development of injury prevention strategies. The authors highlight the most pressing issues for improvement of the quality in injury epidemiology research for disability sport.

## Key Points

**Table:** 

There are a limited, but growing, number of prospective studies assessing sports injury epidemiology within disability sports.
Study quality is variable, such that sports injury definitions, methodologies and injury rates in disability sport are inconsistent, which prevents conclusions being drawn and the development of injury prevention strategies.
There is an urgent need for consensus on sports injury definition and epidemiological research methodology in disability sports.

## Background

A recent report by the World Health Organization (WHO) estimates that 15.6 % of the world population, equivalent to more than one billion people, is living with some form of long-term disability [1]. Although many forms of disability can initiate a sedentary lifestyle for individuals living with disability, opportunities still exist for regular physical activity through sport that will enhance their levels of physical activity (PA) such that they will benefit from wide-ranging positive PA-related health and social outcomes associated with a physically active and sporty lifestyle. Enhanced levels of daily PA can improve overall physical fitness, thus benefitting psychological and social well-being, positively influencing all levels of function and preventing secondary health problems [2]. However, there are specific facilitators, challenges and barriers to participation in sport that are unique to athletes with disability [3].

Sports injuries pose problems for all athletes, but for athletes with disability they often pose additional problems because of the further limitations they can inflict on an already restricted lifestyle. Injured athletes with a disability may find gaining access to emergency and ongoing healthcare services more difficult, and obtaining the appropriate treatment may be even more challenging [4]. Additionally, the consequences of an injury may severely affect their ability to carry out normal activities of daily living [5]. Benjamin Franklin is credited with recognising that “an ounce of prevention is worth a pound of cure” [6], and for those people living with long-term disability, prevention may have even greater importance than for the general population. Whether using the Van Mechelen model [7] or the more recent Finch TRIPP model [8], it is generally agreed that the first stage in sports injury prevention is establishing the extent of the sports injury problem through injury surveillance and epidemiology, so that the subsequent determination of aetiology and mechanisms of injury allow the identification, development and evaluation of preventive measures. Many disabilities will, by their very existence, affect an athlete’s intrinsic and extrinsic sports injury risk factors; for example, athlete collisions in blind football and the grip of a prosthetic limb on a running surface.

For the limited number of researchers working in the specialised but wide field of disability sport, there are further complexities related to the level of an athlete’s disability and the consequent disability classification systems. There are inevitably fewer subjects available to study in disability sports, which leads to important limitations in identifying sufficiently large sample populations for analyses to reach statistically significant research conclusions [9].

The aim of this review is to systematically review the definitions, methodologies and injury rates in disability sport, which should assist the identification and development of injury prevention strategies. A secondary aim is to highlight the most pressing issues for improvement of the quality of injury epidemiology research for disability sport. To our knowledge this is the first systematic review of sports injury within disability sport.

## Methods

This study was conducted in accordance with the Meta-Analysis of Observational Studies in Epidemiology (MOOSE) guidelines for systematic reviews of observational studies [10].

### Information Sources and Search

**Fig. 1:**
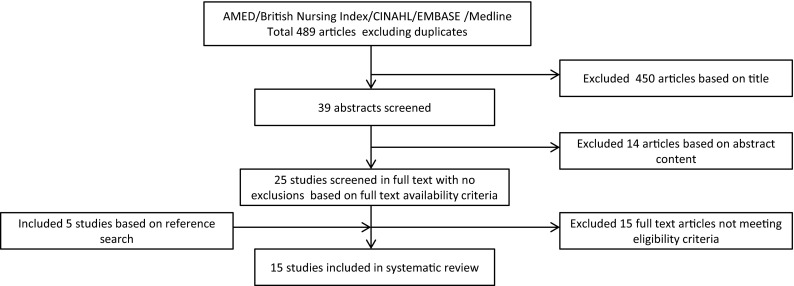
Flowchart of the inclusion process of the articles in the systematic review

An electronic database search was carried out using The National Institute for Health and Care Excellence (NICE) Evidence Healthcare Databases, including the Allied and Complementary Medicine Database (AMED) (1985 to present), British Nursing Index (1992 to present), Cumulative Index to Nursing and Allied Health Literature Database (CINAHL) (1981 to present), Excerpta Medica Database (EMBASE) (1980 to present) and Medline (1946 to present) with no limits on date of publication. The date of the last search by the lead author (RW) was on 16 June 2015. It was decided to use a broad search for the identification of relevant studies limited to English language publications. The literature search therefore used the following keywords: (“athletic injuries”[MeSH Terms] OR “sports medicine”[MeSH Terms]) AND (((paralympic[All Fields] OR paralympics[All Fields]) OR (disability[All Fields] AND (“sports”[MeSH Terms] OR “sports”[All Fields] OR “sport”[All Fields]))) OR parasport[All Fields]) AND “humans”[MeSH Terms]. The search produced 489 results (Fig. 1) and each reference list of the relevant identified articles was crosschecked to confirm that eligible articles were not missed. Eligibility criteria were applied to the screening of titles, abstracts and full texts. Each step in article selection was performed and agreed by two reviewers (RW and EV) without disagreements.

### Eligibility Criteria

The inclusion and exclusion criteria were developed by one author (RW) and agreed by a second author (EV).

#### Type of Study

Only prospective cohort studies were included to minimise errors associated with data recall in retrospective studies [11]. There is a diverse range of definitions for sports injury and a paucity of prospective studies; so all synonyms for sports injury were accepted.

#### Type of Participants

Studies eligible for review included athletes with disability competing and participating in disability sport, without limitation by age, sex, sport or nature of disability. Participants were involved in summer and winter sports and a wide range of competitions and leagues, such as Paralympics, Special Olympics and non-paralympic events.

### Data Extraction and Data Analysis

The lead author extracted the following information from each article, which is included in Table 1: year of publication, title, authors, declared conflicts of interest, ethical approval, use of the STROBE (Strengthening the Reporting of Observational Studies in Epidemiology) checklist (post 2007 studies only), injury definition, exposure (duration of study in days or exposure hours), number of subjects, sport, number of sports injuries and relative risk of injury (IR) per 1000 athlete days with 95 % confidence intervals (CIs). Few studies reported injuries in terms of athlete disability, disability sport classification, nature of injury (for example acute and chronic) or injury severity, so this data was not homogeneous, comparable between studies and therefore not included in Table 1.

**Table 1:** Characteristics of studies

Study	Title	COI	Ethics committee approval	STROBE checklist (for studies after 2007)	Sports injury definition	Injury definition summary	Exposure (days)	Number of subjects	Sports injuries	RRI per 1000 athlete days and 95 % CI
Gawronski et al. 2013 [18]	Fit and healthy paralympians—medical care guidelines for disabled athletes: a study of the injuries and illnesses incurred by the Polish Paralympic team in Beijing 2008 and London 2012	None	Yes	Not reported	Injury and illness defined as any symptom that received medical attention. Injury was defined as a newly acquired musculoskeletal symptom or an exacerbation of a pre-existing (chronic) injury that occurred during training and/or competition	Medical attention	21 (Beijing 2008)	91	57	29.8 (22.1–37.6)
						Medical attention	16 (London 2012)	100	24	15.0 (9.0–21.0)
Magno e Silva et al. 2013 [19]	Sport injuries in elite Paralympic swimmers with visual impairment	Not reported	Yes	Not reported	A reportable injury was defined as any injury that caused an athlete to stop, limit or modify participation for 1 or more days	Time loss ≥1 day	12^a^ (Paralympic Games 2004)	3	4	111.1 (2.2–220.0)^a^
						Time loss ≥1 day	20^a^ (Pan American Games 2005)	23	7	15.2 (3.9–26.5)^a^
						Time loss ≥1 day	13^a^ (IBSA World Championships 2007)	14	22	120.9 (70.4–171.4)^a^
						Time loss ≥1 day	17^a^ (Pan American Games 2007)	13	6	27.2 (4.4–48.9)^a^
						Time loss ≥1 day	12^a^ (Beijing 2008)	3	2	55.6 (0–132.6)^a^
Willick et al. 2013 [20]	The epidemiology of injuries at the London 2012 Paralympic Games	None	Yes	Not reported	Any sport-related musculo-skeletal or neurological complaint prompting an athlete to seek medical attention, regardless of whether or not the complaint resulted in lost time from training or competition	Medical attention	14 (3 days pre-competition and 11 days competition)	3565	633	12.68 (11.7–13.7)
Magno e Silva et al. 2013 [21]	Sports injuries in paralympic track and field athletes with visual impairment	None	Yes	Not reported	A reportable injury was defined as any injury that caused an athlete to stop, limit, or modify participation for 1 or more days	Time loss ≥1 day	12^a^ (Paralympic Games 2004)	11	11	83.3 (34.1–132.6)^a^
						Time loss ≥1 day	20^a^ (Pan American Games 2005)	28	16	111.1 (2.2–220.0)^a^
						Time loss ≥1 day	13^a^ (IBSA World Championships 2007)	28	28	76.9 (48.4–105.4)^a^
						Time loss ≥1 day	17^a^ (Pan American Games 2007)	19	11	34.1 (13.9–54.2)^a^
						Time loss ≥1 day	12^a^ (Beijing 2008)	22	11	41.7 (17.0–66.3)^a^
Magno e Silva et al. 2013 [22]	Sports injuries in Brazilian blind footballers	Not reported	Yes	Not reported	A reportable injury was defined as any injury that caused an athlete to stop, limit, or modify participation for 1 or more days	Time loss ≥1 day	12^a^ (Paralympic Games 2004)	8	12	125.0 (54.3–195.7)^a^
						Time loss ≥1 day	20^a^ (Pan American Games 2005)	8	6	37.5 (7.5–67.5)^a^
						Time loss ≥1 day	13^a^ (IBSA World Championships 2007)	8	7	67.3 (17.5–117.2)^a^
						Time loss ≥1 day	17^a^ (Pan American Games 2007)	8	3	22.1 (0–47.0)^a^
						Time loss ≥1 day	12^a^ (Beijing 2008)	8	7	72.9 (18.9–126.9)^a^
Webborn et al. 2012 [23]	The injury experience at the 2010 Winter Paralympic Games	Declared	Yes	Not mentioned	Any sports-related musculoskeletal complaint that caused the athlete to seek medical attention during the study period, regardless of the athlete ’ s ability to continue with training or competition	Medical attention	17^a^	505	106 injuries (actual injuries reported as 120 but need to remove 14 as states were not sports related)	12.4 (10.0–14.7)^a^
Chung et al. 2012 [24]	Musculoskeletal injuries in elite able-bodied and wheelchair foil fencers—A pilot study	None	Yes	Before STROBE	Injury defined as trauma that occurred during a training/competition and prohibited the athlete from continuing fencing activity for at least 1 day	Time loss ≥1 day	24,664 h^b^	14	95	3.9 per 1000 athlete hours (3.1–4.6)^c^
Ramirez et al. 2009 [25]	Sports injuries to high school athletes with disabilities	No financial relationships relevant to article to disclose	Yes	Not reported	‘Injury episodes’ defined as events resulting in immediate removal of the athlete from the session and medical treatment by school staff or transport to a hospital. ‘Injury diagnoses’ were defined as the physical trauma sustained to the body region of an athlete during the injury event	Medical attention	19,012 h^a,b^	210	38	2.0 per 1000 athlete hours (1.4–2.6)^a,c^
Webborn et al. 2006 [26]	Injuries among disabled athletes during the 2002 Winter Paralympic Games	Not reported	Not reported	Before STROBE	Not reported	Medical attention	20^a^	416	39	4.7 (3.2–6.2)^a^
Sobiecka 2005 [27]	Injuries and ailments of the Polish participants of the 2000 Paralympic Games in Sydney	Not reported	Not reported	Before STROBE	Not reported	Medical attention	23^a^	114	125 injuries to motor system and 1 abrasion/bruise	48.1 (39.7–56.5)^a^
Ferrara et al. 2000 [28]	A longitudinal study of injuries to athletes with disabilities	Not reported	Not reported	Before STROBE	A reportable injury defined as an injury/illness that was evaluated by the US team medical staff during these competitions	Medical attention	13^a^ (1990 World Games and Championships)	220	52	18.2 (13.2–23.1)^a^
							12^a^ (1991 US Paralympic trials)	345	170	41.1 (34.9–47.2)^a^
							24^a^ (1992 Barcelona Paralympics)	360	387	44.8 (40.3–49.3)^a^
							7^a^ (1994 World Athletics Championships)	55	22	57.1 (33.3–81.0)^a^
							14^a^ (1996 Atlanta Paralympics)	380	406	76.3 (68.9–83.7)^a^
Nyland et al. 2000 [29]	Soft tissue injuries to USA paralympians at the 1996 Summer Games	None	Not reported	Before STROBE	Soft tissue injuries operationally defined as strain, sprain, tendonitis, bursitis or contusion	Medical attention	10^a^	304	254 soft tissue injuries	83.6 (73.3–93.8)^a^
Burnham et al. 1991 [30]	Sports medicine for the physically disabled: The Canadian team experience at the 1988 Seoul Paralympic Games	Not reported	Not reported	Before STROBE	Not reported	Assumed medical attention	10^a^	151	84 musculoskeletal conditions treated	55.6 (43.7–67.5)^a^
Robson 1990 [31]	The Special Olympic Games for the mentally handicapped - United Kingdom 1989	Not reported	Not reported	Before STROBE	Not reported	Assumed medical attention	8^a^	1512	127 sport injuries (deduced from treatment summaries)	10.5 (8.7–12.3)^a^
McCormick et al. 1990 [32]	Injury and illness surveillance at local Special Olympic games	Not reported	Yes	Before STROBE	A sports injury was defined as an injury resulting directly from participation in a sports event	Assumed medical attention	3^a^	777	4	1.7 (0–3.4)^a^

*CI* confidence interval,* COI* authors declared conflicts of interest,* IBSA* International Blind Sports Federation,* RRI* relative risk of injury, *STROBE* Strengthening the reporting of observational studies in epidemiology

^a^Deduced or calculated from the study

^b^Exposure in hours

^c^RRIs per 1000 h exposure

Tools for assessing study quality are great in number, yet lack agreement on critical elements and validity for use with sports injury studies assessing injury rates [12, 13]. Therefore, for this review the authors applied a 10-point quality score used in four previous reviews on sports injury outcomes [14–17]. To analyse quality of the selected studies we used the following list of questions:

1. Definition of injury described in each study (yes/no).
2. Studies with prospective designs that presented incidence or prevalence data (yes/no).
3. Description of the population of athletes (e.g. sport, disability, classification) or the player positions (e.g. goalkeepers or forward players) that participated in the study (yes/no).
4. Was the process of inclusion of athletes in the study at random (i.e. not by convenience) or was data collection performed with the entire target population (yes/no).
5. Data analysis was performed with at least 80 % of the athletes included in the study (‘yes’ or ‘no’).
6. Were data regarding the injuries reported by a healthcare professional (yes/no).
7. Was the same mode of data collection (e-mail, telephone, interview, etc.) used (yes/no).
8. Was the diagnosis conducted by medical doctors (yes/no).
9. Was there a follow-up period of at least 6 months for prospective studies (assessed by ‘yes’ or ‘no’).
10. Were the incidence or prevalence rates of injury expressed by a ratio that represents both the number of injuries as well as the exposure to sport (e.g. IR/1000 h of sport exposure, and this criterion was assessed by ‘yes’ or ‘no’).

An answer of yes scored 1 point and no scored zero points resulting in an overall score out of 10 for each study. Two authors (RW and EV) scored quality independently and agreed on all scores; these scores are included in Table 2.

**Table 2:** Quality score assessment of the studies

Study	(1) Definition of injury described in each study (yes/no)	(2) Study with prospective designs that presented incidence or prevalence data (yes/no)	(3) Description of the population of athletes (e.g. sport, disability, classification) or the player positions (e.g. goalkeepers or forward players) that participated in the study (yes/no)	(4) Was the process of inclusion of athletes in the study at random (i.e. not by convenience) or was the data collection was performed with the entire target population (yes/no)	(5) Data analysis was performed with at least 80 % of the athletes included in the study (‘yes’ or ‘no’)	(6) Were data regarding the injuries were reported by a healthcare professional (yes/no)	(7) Was the same mode of data collection (e-mail, telephone, interview, etc.) was used (yes/no)	(8) Was the diagnosis was conducted by medical doctors (yes/no)	(9) Follow-up period of at least 6 months for prospective studies (assessed by ‘yes’ or ‘no’)	(10) Incidence or prevalence rates of injury expressed by a ratio that represents both the number of injuries as well as the exposure to sport (e.g. IR/1000 hours of sport exposure, and this criterion was assessed by ‘yes’ or ‘no’)	Total
Gawronski et al. 2013 [18]	1	1	1	1	1	1	1	1	0	1	9/10
Magno e Silva et al. 2013 [19]	1	1	1	1	1	1	1	1	0	1	9/10
Willick et al. 2013 [20]	1	1	1	1	0	1	0	0	0	1	6/10
Magno e Silva et al. 2013 [21]	1	1	1	1	1	1	1	1	0	1	9/10
Magno e Silva et al. 2013 [22]	1	1	0	1	1	1	1	1	0	1	8/10
Webborn et al. 2012 [23]	1	1	0	0	N/R	1	1	0	0	0	4/10
Chung et al. 2012 [24]	1	1	1	1	0	1	1	0	1	1	8/10
Ramirez et al. 2009 [25]	1	1	1	0	1	0	1	0	1	1	7/10
Webborn et al., 2006 [26]	0	0	1	1	N/R	1	0	0	0	0	3/10
Sobiecka. 2005 [27]	0	1	1	1	1	1	1	0	0	0	6/10
Ferrara et al. 2000 [28]	0	0	0	1	1	1	1	0	0	0	4/10
Nyland et al. 2000 [29]	1	0	1	1	1	1	1	0	0	0	6/10
Burnham et al. 1991 [29]	0	1	0	1	1	1	1	0	0	0	5/10
Robson. 1990 [30]	0	1	0	1	1	1	1	0	0	0	5/10
McCormick et al. 1990 [31]	1	1	0	1	1	0	1	1	0	1	7/10

1: yes, 0: No, *N/R* not reported, *IR* injury risk

In order to compare injury risk in disability sports, where possible, injury data were extracted and dates for data collection were used to calculate 95 % CIs. Where the number of days or specific dates were not mentioned in study methods but the study suggested that the duration of the competition (not including pre-competition) was the same as the duration of data collection, the 95 % CI was calculated using the competition dates found from internet sources. Injury risk and 95 % CIs were only compared between studies with comparable injury definitions. Where a study included injury data for separate competitions with long time intervals between the individual competitions, injury risk was calculated for each competition, rather than pooling data: this allowed comparison of results with other short-duration competition-based studies.

## Results

### Identification of Studies

The initial search yielded 489 potentially relevant papers following removal of duplicates. The study identification procedure and flow chart are included in Fig. 1. Following removal of studies not matching the inclusion criteria based on the title, 39 papers remained. The abstracts from these papers were independently evaluated by two of the authors (RW and EV), which further reduced the number of relevant studies to 25. No studies were excluded on full-text availability criteria. The reference lists of the 25 papers were read (RW and EV), which identified five further studies. After reading all 30 papers in full, 15 studies were excluded, which resulted in 15 studies being included in the systematic review [18–32]. However, 13 studies had injury reported by a healthcare professional. Owing to the wide range of study methodologies adopted in the 15 studies, data could not be pooled for analysis.

Table 1 demonstrates the increased number of prospective epidemiological studies covering sports injuries in disability sport in the last 3 years (seven studies; 47 %) compared to the preceding 22 years (eight studies; 53 %).

### Description of the Included Studies

It is interesting to observe the increase in number of prospective epidemiological studies covering sports injuries in disability sport in the last 3 years (seven studies; 47 %) compared to the preceding 22 years (eight studies; 53 %), and that most prospective studies are published following the Paralympic Games (Table 1), with a particularly large spike following the London 2012 Summer Paralympic Games [33]. Table 1 demonstrates that the majority of studies referred to short competitions, Paralympic Games and a wide variety of summer and winter sports. Of the 15 studies, only two were longitudinal with follow-up beyond 6 months and only six studies explicitly had injury diagnosis confirmed by a medical doctor and/or physiotherapist. When studies are assessed by participant numbers (Table 1), the number of participants ranged from 13 [22] to 3565 [20] (mean 291; median 28); however 38 % of all participants were from the 2012 summer Paralympic Games study [20]. Of the 11 studies that reported athlete sex, 68 % of participants were male and 32 % were female.

Athlete sport classification [34], which is a grading system for competitor disability and resultant sport-specific functional impairment, was reported in only three studies; these studies were all by the same lead author and referred to competitions involving Brazilian national teams with visual impairment (swimming, track and field athletics, and football) [19, 21, 22]. The athletes are classified by an ophthalmologist into three categories: B1 or S11 in swimming (from no light perception in either eye to light perception, unable to recognise the shape of a hand at any distance or direction); B2 or S12 in swimming (from ability to recognise the shape of a hand up to a visual acuity of 20/600 or a visual field of less than 5° in the best eye with the best practical eye correction); B3 or S13 in swimming (from visual acuity above 20/600–20/200 or a visual field of less than 20° and more than 5° in the best eye with the best correction) [35]. In football 5-a-side (also known as blind football), only B1 athletes compete, and to ensure fairness of competition for those with some vision, blindfolds are worn to cover the eyes.

Table 1 shows there is a lack of consistency in reporting across all injury parameters. For example, the number of studies using the following injury definitions: medical attention: eight (53 %); time loss ≥1 day: four (27 %); not specifically defined but implied medical attention: three (20 %), and five of these studies (33 %) provided no clear definition of a sports injury. A similar level of inconsistency exists for the number of studies reporting athlete exposures with days exposed: eight (53 %); hours exposed: two (13 %); competition days: four (27 %); inferred to be the duration of the competition: one (7 %). No studies reported information on injury severity. For injury diagnoses, seven studies (47 %) reported broad diagnoses of an injury, but there was no consistency in the diagnosis system used; when an anatomical site of injury was reported no two studies used the same methods. Ten studies (67 %) reported injuries by specific disability, but only three (20 %) included athlete classification. Baseline data for study participants was limited, with only five studies (33 %) reporting athletes’ mean age and 11 studies reporting athlete gender (73 %).

### Injury Rates in Disability Sports

Table 1 demonstrates the wide variability in reported injury risk across different studies. Only two studies reported IRs per 1000 athlete days with 95 % CIs and one study reported IR per 1000 h exposure; all other values included in this review were calculated from data included in the original paper. It was not possible, however, to calculate IR values per 1000 athlete days for two studies, as the authors reported exposure in hours. For those studies where injuries were reported by medical attention injuries, the studies with larger sample populations appear to report a lower injury risk.

## Discussion

Given the heterogeneous nature of the published studies on disability sport and the variations found in study methods, it is not possible to draw conclusive findings about the epidemiology of injuries in disability sport. This heterogeneity may, in part, be explained by the general evolution of sports injury research methods over the 25 years spanning the studies [16]. Further barriers to meaningful conclusions include the following: a small number of studies were identified with wide subject heterogeneity and a large number of sports; only two studies had a duration beyond 6 months and the remainder covered short competitions, or a series of short competitions; and none of the studies considered the importance of injury severity and therefore comparisons of injury severity within different disability sports and with able-bodied athletes were not possible.

There have been two recent non-systematic reviews covering disability sport. Fagher and Lexell [36] identified ten relevant prospective studies, whereas we identified 15, notwithstanding the time lag until our review. Of the 15 retrospective studies included in their review, seven did not report injury definitions, and injuries included in the review varied from ‘athlete concerns’ to major trauma; in addition recall times were up to 1 year post-injury. Injury definitions were of such poor quality in the retrospective studies that they did not inform the review and perhaps more importantly there was no systematic assessment of reported injury rates in the paper. Webborn and Emery [37] included 17 studies without identifying whether study data were collected retrospectively or prospectively and did not review definition of injury. The latter review was specifically restricted to Paralympic sports and one of their included studies was not a peer-reviewed article. Furthermore, as with non-disability sports [38], the risks of injury may vary between different sports, but for disability sports they may also vary within disability classifications within each sport, which limits the value of making comparisons within and across sports with respect to developing injury prevention protocols.

The most important conclusion obtained from this review therefore is the identification of an urgent need for a consensus to be developed on definitions and methods used for conducting and reporting epidemiological studies in disability sports. In particular, consideration must be given to standardising reporting parameters such as disability, impairment (classification where appropriate), exposure, injury definitions, injury coding (both nature and anatomical site), severity and return to fitness criteria following injury. In addition, criteria for differentiating between acute and gradual onset sports injuries and the deterioration in an athlete’s existing chronic conditions is an important factor in disability sport.

In order to better inform and improve future study quality in injury epidemiology research for athletes with disability, the authors have identified the most pressing reporting issues reported in Table 3. Table 3 includes a mixture of basic methodological omissions found in current papers and application of lessons learnt from non-disability sports epidemiological studies. Consideration of identifiable issues can lead to potential solutions for future studies and Table 3 is not intended to be exhaustive or prescriptive, but should help researchers improve the quality of injury epidemiology data leading to better-designed longitudinal studies. Intrinsic baseline data have not been consistently reported to date and extrinsic risk factors have not been reported at all.

**Table 3:** Pressing issues to improve the quality in injury epidemiology research for disability sport

Study methodology	Intrinsic athlete baseline data	Extrinsic athlete data
*Pressing issues deduced from the current review*		
Reporting of the employed sports injury definition, preferably using a standardised injury definition Report on whether an injury diagnosis was made by medical professional, and include details whether diagnosis was confirmed with objective methods Use of standard terminology of injury diagnosis (e.g. The Orchard Sports Injury Classification System) Use of a prospective study design Reporting whether an injury was sustained in training or competition Categorising injuries into acute and chronic (i.e. overuse) Reporting on the mechanism of injury Reporting of sporting exposure, ideally in hours of play Reporting of proper injury numbers, i.e. incidence (density) and /or prevalence of injury	Reporting of basic cohort demographics: **•** Age **•** Sex **•** Type of sport **•** Disability category **•** Disability severity **•** Athlete classification (if applicable)	
*Issues deduced from applying practical experience to review findings*		
Requirement for valid and reliable clinical tests to accurately determine diagnoses in disability athletes	**•** Reporting on whether the disability is congenital or acquired, and if acquired include duration since acquisition **•** Provision of regular medication use **•** Reporting on current and previous treatment for disability or sports injury **•** Prospective monitoring of (changes in) training load	Prospective reporting of: **•** Equipment use for sport **•** Equipment use to support disability **•** Sporting surface **•** Climate conditions **•** Level of competition

Study quality assessment scores varied widely between the studies included in the review and this reflects the range in the quality of published sports injury research in disability sport. Considerable caution must be used when interpreting the risk of bias assessment shown in Table 2, as this does not necessarily reflect study quality. All tools for quality assessment in systematic reviews of observational cohort studies are fraught with limitations, which is why no single tool can generically and reliably assess study quality or bias [12]. While the studies included in this review may provide important information for those people planning the medical logistics (e.g. staff and equipment) required to support disability sports competitions, if injury risk is to be better understood and risk factors are to be determined so that injury prevention models can be explored, greater consistency and higher standards are required in study methodology. This would make disability sports injury studies more comparable and open to pooling of data in the future. Furthermore, intrinsic and extrinsic risk factor data have not been reported to date in disability sport, events and athletes are fewer in number, and multicentre data studies do not exist allowing for wider data collection, which pose considerable challenges to research and knowledge development.

Another fundamental issue encountered during the review relates to the use of different definitions for sports injury. For those studies offering a definition, injury definitions vary from any conditions involving a medical consultation with a healthcare professional, without consideration of outcomes, to conditions resulting in 1 or more days’ absence from training or competition. Much has been written on the optimal definition of sports injury and nuances between sports [11, 39–41], but there has to date been no consideration of applications to disability sports. Studies reporting a sports injury definition utilised ‘time loss’ or ‘medical attention’, which suggests that the reported injuries are primarily acute, including traumatic injuries, and that overuse injuries may be under-reported even though they were largely indeterminable from data presented in the studies. Thirteen papers reported injuries at short competitions (range 3–23 days), which further supports the notion of emphasis towards competition-based acute injury inclusion and overuse injury exclusion, as training injuries have consistently been overlooked, owing to a lack of longitudinal studies [18–23, 26–32]. A recent pilot study by Clarsen et al. [41] utilised inclusion of all physical complaints regardless of their consequences, which allows improved identification of risk factors for injury by placing sports injuries in greater intrinsic and extrinsic context: this approach may therefore be more applicable to athletes with disability, whose chronic physical conditions make them eligible to choose to play and compete in sports for people with disability. A potential risk factor within disability sport is the disability itself and the severity of disability. However, the importance of the type and level of disability on sports injury risk remains unknown. Paradoxically, while a severe impairment could have a negative effect on an athlete’s sporting performance (e.g. speed, agility, distance covered, acceleration and deceleration) when compared to a milder impairment, it could therefore potentially reduce injury risk in certain sports.

When disability or classification groups were reported the participant numbers became so low that injury risk conclusions became even more uncertain. For elite competitive sport, classification is complex, differs for each disability and can vary even across sports for the same athlete. In grassroots and non-competitive sports the processes of determining eligibility and classification may not be as robust and these issues make epidemiological research for athletes with disability challenging. Studies analysing disability sports injury will, on the very basis of sub-categorisation, result in small sub-group numbers, which pose challenges in determining statistical significance and study power, notwithstanding differences between sports, disability, classification and athlete position. However, this must not be used as a reason to accept lower research standards, which are expected within comparable non-disability sport studies. Very few studies collected and, therefore, reliably reported actual duration of sport exposure, which limits comparability of injury risk between sports and studies, the identification of risk factors, incidence rates and injury prevention efficacy.

Study data were determined by individuals reported as being orthopaedic surgeons, physiotherapists, nurses and coaches with no further information provided on the experience or training of the attending personnel. The validity and reliability in diagnosis of sports injury is therefore a source of uncertainty, as only 13 studies (87 %) report sports injury diagnosis by healthcare professionals, although five (33 %) of these were by doctors. To our knowledge, no accepted clinical tests for sports injury have been validated in disability populations, which raises important questions about the sensitivity and specificity of injury diagnoses in disability sport. Some paralympic games studies mentioned the use of radiological imaging, which could confirm or refute some of these diagnoses, but confirmation of diagnoses with radiological findings was not reported in data.

## Conclusions

There are a limited, but growing, number of prospective studies assessing sports injury epidemiology within disability sports. The quality of studies is variable, such that sports injury definitions, methodologies and injury rates in disability sport remain inconsistent, which prevents conclusions being drawn and the development of injury prevention strategies. Key issues include lack of conformity on sports injury definitions, lack of consensus on methodology and reporting for disability sports injury studies, disability and impairment descriptor reporting omissions, focus on short-term competition-based studies, lack of long-term follow-up, athlete baseline data rarely being collected, consistency of exposure reporting and injury severity not being reported.

The authors highlight the most pressing issues to improve the quality in injury epidemiology research for disability sport in Table 3. Without addressing methodological improvements suggested in this review, the development of injury prevention strategies for athletes with disability will remain elusive, as injury surveillance will not be able to establish the extent of the sports injury problem, which is the first step in sports injury prevention.
